# Detection of Cardiotoxicity Using Right Ventricular Free Wall Longitudinal Strain in Low Cardiovascular Risk Breast Cancer Patients Receiving Low-Dose Anthracycline Treatment

**DOI:** 10.7759/cureus.63138

**Published:** 2024-06-25

**Authors:** Nana Gorgiladze, Mikheil Shavdia, Tamar Gaprindashvili, Elene Gogua, Lika Gachechiladze, Mata Gujabidze, Zurab Pagava

**Affiliations:** 1 Department of Clinical Oncology, Tbilisi State Medical University, Tbilisi, GEO; 2 Department of Cardiopulmonology, Bokhua Memorial Cardiovascular Center, Tbilisi, GEO; 3 Department of Oncology, Institute of Clinical Oncology, Tbilisi, GEO; 4 Department of Cardiology, Meta Clinic, Tbilisi, GEO

**Keywords:** right ventricle, global longitudinal strain (gls), cardiotoxicity, anthracycline, 2d echocardiography

## Abstract

Objective

Breast cancer patients who receive chemotherapy may develop cancer therapy-related cardiovascular toxicity, particularly if they have pre-existing cardiovascular risk factors. Notably, right ventricle dysfunction may manifest before the left ventricle. Our study aims to compare conventional echocardiography with global longitudinal strain (GLS) in low cardiovascular risk patients on low-dose anthracycline, focusing on early cardiotoxicity detection. Additionally, we explore the predictive role of right ventricular free wall longitudinal strain (RVFWLS) in cardiotoxicity.

Methods

In a recent study, 28 women with low cardiovascular risk who underwent low-dose anthracycline chemotherapy for breast cancer were assessed for cardiac function using two-dimensional echocardiography and speckle-tracking echocardiography. The measurements included left ventricular ejection fraction (LVEF), right ventricular systolic function (RVS'), tricuspid annular plane systolic excursion (TAPSE), left ventricular global longitudinal strain (LVGLS), and RVFWLS. All patients had normal LVEF at the beginning of the study. Cardiotoxicity was defined as a new decrease in LVEF by 10% or below 53% and/or changes in LVGLS/RVFWLS by 15%.

Results

In our study, no significant changes were observed in the LVEF following chemotherapy treatment. The LVEF values remained stable, changing slightly from 63 ± 3.7 to 65.0 ± 3.4, with a t-test value of 1.790 and a p-value of 0.079. Similarly, the analysis found no significant changes in RVS' and TAPSE values following chemotherapy treatment. However, significant changes were observed in strain measurements. LVGLS decreased from -21.2 ± 2.1 to -18.6 ± 2.6 (t-test = -4.116; df = 54, p=0.001), and RVFWLS decreased from -25.2 ± 2.9 to -21.4 ± 4.4 (t-test = -3.82; df = 54, p=0.001). Notably, 35% of participants showed changes in RVFWLS greater than 15%, whereas LVGLS changed by less than 15%. This indicates that RVFWLS is more sensitive to the treatment compared to LVGLS.

Conclusions

The study results indicate that during the initial phases of chemotherapy treatment in low cardiovascular risk patients, early changes in strain measures reveal subclinical cardiotoxicity. This suggests that GLS measurements are more effective at detecting early signs of myocardial damage and potential deterioration in cardiac function than traditional echocardiographic parameters. Additionally, it is noteworthy that RVFWLS exhibits greater sensitivity to these changes, regardless of the chemotherapy dosage and regimen.

## Introduction

Breast cancer, a global concern affecting women, has seen increased incidence but reduced mortality, credited to early detection and treatment advances [1-3]. However, these treatments reveal diverse chemotherapy-related side effects [4]. Anthracyclines are drugs used to treat breast cancer by interrupting DNA replication [5,6], but they also cause heart muscle damage, leading to cardiotoxicity, which is a serious concern. High doses of anthracyclines or pre-existing heart disease increase the risk of cardiotoxicity, characterized by a decline in left ventricular ejection fraction (LVEF) [7,8]. Early detection and intervention are crucial to prevent further cardiac damage and improve patient outcomes.

Echocardiography is recommended as the method of choice for detecting cancer therapy-related cardiovascular toxicity (CTR-CVT). It assesses both systolic and diastolic functions of the left and right ventricles, volumes, and dimensions [9]. LVEF is a robust prognostic predictor for assessing left ventricular function, though it exhibits low sensitivity to subtle changes in contractile function [10]. In the latest ESC guidelines on cardio-oncology, global longitudinal strain (GLS) is essential in diagnosing oncological therapy's cardiotoxicity [11,12]. A relative drop in GLS of 15% is the suggested threshold for diagnosing subclinical heart impairment [13].

Several studies indicate that right ventricular (RV) remodeling occurs concurrently with left ventricular (LV) remodeling and that the LV and RV deformation mechanics follow similar temporal patterns and degrees of impairment during chemotherapy treatment [14]. As a result, RV remodeling should be noticed in the overall cardiac assessment of chemotherapy patients, particularly those receiving anthracyclines [15]. However, due to its complex morphology (compared to the LV's ellipsoidal shape, the RV appears triangular from the side and crescent-shaped in cross-section), complex systolic motion, and prominent myocardial trabeculations, measuring RV systolic function (RVS') is limited and difficult in routine clinical practice. Thus, there is a scarcity of data for parameters that may reflect subclinical RV dysfunction and may predict RV cardiotoxicity [16-18].

Natriuretic peptides are crucial biomarkers for pressure overload and myocardial stretch, and their capacity to identify hemodynamic stress makes them essential for the detection of cardiotoxicity as well as for long-term monitoring in the treatment of heart failure [19,20].

Our study aims to examine the predictive role of the GLS techniques in patients receiving low-dose anthracycline treatment and low cardiovascular risk, as well as evaluate structural and functional changes of the RV during chemotherapy.

This article was previously posted to the Authorea preprint server on October 29, 2023 (DOI: 10.22541/au.169858263.30353223/v1)

## Materials and methods

Inclusion and exclusion criteria

The study included female patients aged 18-70 years with newly diagnosed breast cancer who were scheduled to receive an anthracycline chemotherapy regimen. Exclusion criteria were refusal to participate in the trial, a prior diagnosis of coronary artery disease or heart failure with an LVEF <55%, previous chemotherapy treatment, and age over 70 years.

Study population

Women with breast cancer were assessed according to the HFA-ICOS risk assessment (cardiovascular risk factors, cardiovascular disease history, cancer history, and cancer treatment history). Thirty-two patients with low cardiovascular risk (defined as having no risk factors or one moderate risk factor (1 point, according to the 2022 HFA-ICOS baseline cardiovascular toxicity risk stratification) who had planned anthracycline chemotherapy for breast cancer were prospectively included in this single-center study between June 2022 and August 2023. Four patients were excluded due to poor two-dimensional (2D) image quality. As a result, 28 patients were enrolled in the trial at the end. The study received ethical approval from the Tbilisi State Medical University Biomedical Research Ethics Committee (approval number: N3-2021/87; approval date: March 23, 2021, 21:00). All participants provided written informed consent before being included in the trial.

Chemotherapy regimen

Patients received either a dose-dense AC (doxorubicin 60 mg/m2, cyclophosphamide 600 mg/m2, paclitaxel 175 mg/m2) or EC (epirubicin 100 mg/m2, cyclophosphamide 830 mg/m2) chemotherapy regimen with an inter-cycle interval of 14-21 days. Demographic data, echocardiographic parameters, and cardiac biomarkers were documented. 2D echocardiography, electrocardiography, and serum cardiac N-terminal portion pro-natriuretic peptide type B (NT-pro BNP) were routinely performed at baseline before the chemotherapy (T0) and at the end of the treatment (fourth cycle, T1).

Standard echocardiographic data

All patients underwent standard transthoracic 2D echocardiography conducted by the same investigator during both visits (T0-T1) using a GE Vivid E9 ultrasound machine (GE Healthcare, Horten, Norway) equipped with an M5S (1.7-3.3 MHz) transducer, following the American Society of Echocardiography guidelines (ASE). All echocardiographic images were acquired on average at 70-90 frames per second and digitally stored for three cardiac cycles. LVEF was measured in apical four- and two-chamber views using the biplane Simpsons method, with three consecutive heart cycles recorded for each view. ASE standards were used to obtain septal and posterior wall thicknesses, LV, aorta, and left atrial diameters. The RV-focused apical four-chamber view was used for echocardiographic RV function measurements. Tricuspid annular plane systolic excursion (TAPSE) was defined as the vertical displacement of the tricuspid annulus from end-diastole to end-systole using M-mode. The tissue Doppler-derived tricuspid lateral annular systolic velocity wave (S wave) was obtained by aligning the basal segment and the tricuspid annulus with the Doppler cursor.

Speckle tracking echocardiography

All of the patients were in sinus rhythm. In the LV, the peak-systolic strain was calculated automatically from the mean of the six traced segments for each 2D apical view (two-, three-, and four-chamber). In contrast, LV global longitudinal strain (LVGLS) was calculated by averaging the peak-systolic strain of apical views. The standard RV-focused apical four-chamber view calculates RV free wall longitudinal strain (RVFWLS). The new relative decline in GLS by >15% from the baseline was considered CTR-CVT. The tracking quality was visually validated. Segments that did not track correctly at first were manually adjusted. After manual adjustment, segments that could not be tracked correctly were rejected. Images with poor quality that made speckle analysis impossible in two or more consecutive segments were excluded.

Biomarkers

In our study, the concentrations of NT-pro BNP were measured using a commercially available chemiluminescence immunoassay on a Snibe Maglumi 800 (Shenzhen New Industries Biomedical Engineering Co., Ltd., National Biological Industry Park (Shenzhen, China)) analyzer. The 99th percentile reference limit for NT-pro BNP greater than 125 pg/mL was considered elevated.

Blood samples corresponding to baseline and follow-up time points were included for analysis. Biomarker assessments were routinely performed at baseline and immediately or 24 hours after cumulative dosages of doxorubicin 240 mg/m2 and epirubicin 800 mg/m2 during the follow-up time.

Statistical analysis

Statistical analyses were performed using SPSS Statistics version 23.0. (IBM Corp. Released 2015. IBM SPSS Statistics for Windows, Version 23.0. Armonk, NY: IBM Corp.). Continuous variables are presented as the mean ± standard deviation (SD). A paired t-test (two-sided) was used to compare the studied parameters during observation. A correlation analysis was performed using the Pearson r coefficient. P-values of <0.05 were considered statistically significant.

## Results

At the end of the study, 28 women were included in the final analyses. The mean age of the patients was 48.6 years. The mean cumulative doxorubicin dose was 240 mg/m2 and epirubicin 800 mg/m2. The mean blood pressure was 123/80 mmHg, and the mean heart rate was 80.9 bpm. The baseline characteristics of the patients are listed in Table 1.

**Table 1:** Patients' characteristics

Variables	Mean ± SD	Range
Age	48.6 ±11.8	30-70
Body mass index (kg/m^2^)	29.9 ± 6.62	21.3-46.1
Systolic blood pressure (mmHg)	123.25 ± 20.6	90-140
Diastolic blood pressure (mmHg)	80 ± 11.4	70-100
Heart rate (bpm)	80.9 ± 12.9	60-110

Effects of anthracycline chemotherapy on echocardiographic parameters

Echocardiographic characteristics are listed in Table 2.

**Table 2:** Echocardiographic parameters Data is expressed as mean ± SD, * p<0.05 statistically significant result LVEF: left ventricular ejection fraction; LVGLS: left ventricular global longitudinal strain; RVFWLS: right ventricular free wall longitudinal strain; RVS': right ventricular systolic function; TAPSE: tricuspid annular plane systolic excursion

Variables	Baseline ± SD	At the end of chemotherapy ± SD	p-value
LVEF (%)	63.3 ± 3.7	65 ± 3.4	0.079
LVGLS	-21.2 ± 2.1	-18.64 ± 2.6	<0.001 *
RVFWLS	-25.2 ± 2.9	-21.44 ± 4.4	<0.001 *
TAPSE (mm)	-24.4 ± 3.0	-25.39 ± 2.5	0.181
RVS' (cm/s)	11.00±7	14.3 ± 2	0.152

Impact on the Conventional Parameters

No significant changes were observed in the LVEF after the chemotherapy treatment. The LVEF values remained unchanged from 63.3 ± 3.7 to 65.0 ± 3.4 (with a t-test value of 1.790 and a p-value of 0.079). The ΔLVEF was found to be 1.8 ± 2.6.

Similarly, the analysis found that changes in the values of RVS’ and TAPSE after the chemotherapy treatment were not significant. The RVS' values changed from 0.12 ± 0.07 to 0.14 ± 0.02 (t-test value of 1.454 and a p-value of 0.152), while the TAPSE values changed from 24.4 ± 3.0 to 25.4 ± 2.5 (t-test value of 1.355 and a p-value of 0.181). The ΔRVS’ was found to be 0.03 ± 0.06 and ΔTAPSE 1.0 ± 3.5.

Impact on the GLS

On the other hand, the study's results indicate a significant decrease in the LVGLS and RVFWLS from baseline to the end of the chemotherapy. The LVGLS decreased from -21.2 to -18.6 (t-test = -4.116; df = 54, p=0.001). The change in value ΔLVGLS was 2.6. The mean percentage change of the LVGLS was 11.6% (p=0.001).

Similarly, RVFWLS decreased from -25.2 to -21.4 (t-test = -3.82; df = 54, p=0.001). The ΔRVFWLS value of change was 3.8. Likewise, the percentage decrease in the RVFWLS was 15% (p=0.001).

Correlation Between Different Parameters

The study's findings suggest no significant correlation between the changes in LVGLS and the changes in other variables such as RVFWLS, RVS', TAPSE, and LVEF (Table 3).

**Table 3:** LVGLS correlation with other variables LVGLS: left ventricular global longitudinal strain; RVFWLS: right ventricular free wall longitudinal strain; RVS': right ventricular systolic function; TAPSE: tricuspid annular plane systolic excursion, LVEF: left ventricular ejection fraction

LVGLS vs.	LVEF	RVFWLS	RVS'	TAPSE
Pearson r	-0.094	-0.186	0.255	-0.249
p-value	0.634	0.343	0.190	0.201

The data analysis revealed no significant correlation between the change in RVFWLS and the changes in other variables, namely RVS', TAPSE, and LVEF. These findings suggest that RVFWLS may be a unique measure of RV dysfunction at the early stages of chemotherapy (Table 4).

**Table 4:** Correlation between RVFWLS and RVS’, TAPSE, and LVEF LVEF: left ventricular ejection fraction; RVFWLS: right ventricular free wall longitudinal strain; RVS': right ventricular systolic function; TAPSE: tricuspid annular plane systolic excursion

RVFWLS vs.	RVS’	TAPSE	LVEF
Pearson r	-0.163	-0.110	-0.136
p-value	0.407	0.577	0.490

Correlation of Percentage Changes

In comparison to absolute value changes, the percentage change in LVGLS (11.6% ± 13.3%; p=0.001) did not correlate significantly with the percentage change in RVFWLS, TAPSE, or LVEF; however, the negative correlation with RVS' was significant (r = -0.43; p=0.021) (Table 5).

**Table 5:** Correlation of percentage changes of LVGLS with other variables LVGLS: left ventricular global longitudinal strain; RVFWLS: right ventricular free wall longitudinal strain; RVS': right ventricular systolic function; TAPSE: tricuspid annular plane systolic excursion. LVEF: Left ventricular ejection fraction

LVGLS vs.	RVFWLS	RVS'	TAPSE	LVEF
Pearson r	-0.183	-0.433	-0.169	0.103
p-value	0.343	0.021	0.201	0.190

The percentage change of RVFWLS (15,0%, p=0.001) did not correlate significantly with the percentage changes of other variables: ΔRVS', ΔTAPSE, and ΔLVEF (Table 6).

**Table 6:** Correlation of percentage changes of RVFWLS with other variables RVFWLS: right ventricular free wall longitudinal strain; RVS': right ventricular systolic function; TAPSE: tricuspid annular plane systolic excursion; LVEF: left ventricular ejection fraction

RVFWLS vs.	RVS'	TAPSE	LVEF
Pearson r	-0.279	0.126	0.120
p-value	0.150	0.523	0.543

Effects of anthracycline chemotherapy on cardiac biomarker

The study findings show that NT-pro BNP concentration changed at the end of the chemotherapy (t-test = 1.313; df = 54, p=0.195; ΔNT-pro BNP=18.46 ± 79.66) (Table 7). However, despite the tendency to rise, the results were nonsignificant, and there was no correlation between the GLS and NT-pro BNP changes, probably due to the small sample size.

**Table 7:** Cardiac biomarker concentration changes

Cardiac marker	Baseline ± SD (pg/mL)	Follow-up ± SD (pg/mL)	p-value
NT-pro BNP	93.73 ± 96.31	112.2 ± 79.66	0.195

## Discussion

The main challenge for oncology and cardiology is cancer therapy-induced cardiovascular toxicity. The goal of our study was to look for early signs of cardiotoxicity, primarily structural and functional changes in the RV, during breast cancer chemotherapy.

We compared conventional echocardiographic parameters and more precise speckle-tracking echocardiography for this purpose. Under the most recent ESC guidelines on cardio-oncology, the GLS is a crucial component in identifying the cardiotoxicity of oncological therapy. The GLS is a critical aspect in determining cancer treatment cardiotoxicity. The proposed cut-off point for suspecting subclinical heart impairment is a relative drop in GLS of 15% during cancer treatment. Early detection of asymptomatic cardiotoxicity enables the start of cardioprotective therapy. It lowers the possibility that the oncological therapy will be interrupted or altered if the LVEF declines, which may have an impact on survival [21].

Our study's findings revealed that in low cardiovascular risk patients receiving a low-dose anthracycline regimen, measurements of RVS' and TAPSE at the end of treatment were insignificant, and the LVEF remained unchanged. However, there was a significant decrease in both LVGLS and RVFWLS from baseline to the end of the chemotherapy treatment. Notably, significant RVFWLS changes greater than 15% were observed in 35% of participants (10 individuals), while the same patients exhibited LVGLS changes of less than 15%. This decrease suggests that the heart's functional capacity was compromised during chemotherapy and that RVFWLS was more sensitive to the effects of the treatment compared to LVGLS.

Over the past few years, a growing body of evidence has highlighted the importance of RVGLS in the early detection of cardiotoxicity. For example, a study by Zhao et al. found that patients with lymphoma treated with doxorubicin experienced RV remodeling and functional impairment before any LVEF or LV volume changes were observed [18]. Similarly, Arciniegas Calle et al. showed that early GLS changes can predict cardiotoxicity development in patients with anthracycline and trastuzumab [22]. Another study by Boczar et al. reported that RVFWLS decreased significantly in patients with breast cancer who were treated with anthracyclines, which was consistent with the results of other studies [23]. Laufer-Perl et al. also found that anthracycline therapy was associated with a reduction in RV strain values in patients with breast cancer, despite no change in LV function [24]. The studies indicate that RVFWLS is an effective method for detecting cardiotoxicity early on. Moreover, the results of our study suggest that even with low doses of anthracycline, RVFWLS is more sensitive to treatment than LVGLS. This finding is significant as it allows for timely intervention and improved patient outcomes. These observations could be attributed to the RV's anatomical peculiarities: the thinner shape, fewer myofibrils, and greater susceptibility to injury from chemotherapy. This may be one potential cause of RV impairment in patients after anthracycline therapy [25]. There are, however, few data points on variables that can indicate RV subclinical dysfunction and serve as possible RV cardiotoxicity predictors [18]. Furthermore, while the absence of a link between RVFWLS, RVS', and TAPSE is noteworthy, it does not necessarily indicate a lack of clinical value for RVFWLS. Further research is needed to fully understand the clinical implications of these findings.

Although there was some tendency for the cardiac biomarker to be essential to monitor heart function and detect the likely development of heart failure, GLS measurements may provide a more accurate assessment of cardiac function during chemotherapy treatment, allowing for earlier detection of potential cardiac damage and prompt intervention. This could improve patient outcomes and reduce the risk of long-term cardiac complications.

Study limitations

It is crucial to acknowledge that the study has certain constraints, such as a limited number of participants and a brief duration of follow-up. However, the findings offer a significant understanding of the progressive alterations in RVFWLS and emphasize the necessity of closely monitoring cardiac functionality in individuals receiving anthracycline treatment.

## Conclusions

The study's findings suggest that relatively new imaging technique GLS measurements are more sensitive than conventional echocardiographic parameters (LVEF, TAPSE, RVS') for the detection of myocardial damage and a potential decline in cardiac function at the early stages of chemotherapy treatment, which may have implications for patient management and treatment strategies. Moreover, RVFWLS is more sensitive to these changes, despite the dosage and regimen of the chemotherapy. While more research with larger sample sizes and extended follow-up periods is needed to understand the clinical significance of RVFWLS, these results contribute to a growing body of research on RVFWLS and its potential as a clinical tool for evaluating cardiac function. It is important to note that despite the treatment regimen and the patient's cardiovascular risk, precise follow-up is essential to monitor heart function and detect the likely development of heart failure. GLS measurements may provide a more accurate assessment of cardiac function during chemotherapy treatment, allowing for earlier detection of potential cardiac damage and prompt intervention. This could improve patient outcomes and reduce the risk of long-term cardiac complications.

## References

[1] Zhang J, Lu Y, Zhang N (2023). Global burden of female breast cancer and its association with socioeconomic development status, 1990-2044. Cancer Rep (Hoboken).

[2] Bray F, Ferlay J, Soerjomataram I, Siegel RL, Torre LA, Jemal A (2018). Global cancer statistics 2018: GLOBOCAN estimates of incidence and mortality worldwide for 36 cancers in 185 countries. CA Cancer J Clin.

[3] Giaquinto AN, Sung H, Miller KD (2022). Breast cancer statistics, 2022. CA Cancer J Clin.

[4] Alexandre J, Cautela J, Ederhy S (2020). Cardiovascular toxicity related to cancer treatment: a pragmatic approach to the American and European cardio-oncology guidelines. J Am Heart Assoc.

[5] (2005). Effects of chemotherapy and hormonal therapy for early breast cancer on recurrence and 15-year survival: an overview of the randomised trials. Lancet.

[6] Ewer MS, Ewer SM (2015). Cardiotoxicity of anticancer treatments. Nat Rev Cardiol.

[7] Cascales A, Pastor-Quirante F, Sánchez-Vega B (2013). Association of anthracycline-related cardiac histological lesions with NADPH oxidase functional polymorphisms. Oncologist.

[8] Armenian SH, Armstrong GT, Aune G (2018). Cardiovascular disease in survivors of childhood cancer: insights into epidemiology, pathophysiology, and prevention. J Clin Oncol.

[9] Andersson AE, Linderholm B, Giglio D (2021). Delta NT-proBNP predicts cardiotoxicity in HER2-positive breast cancer patients treated with trastuzumab. Acta Oncol.

[10] Khouri MG, Douglas PS, Mackey JR, Martin M, Scott JM, Scherrer-Crosbie M, Jones LW (2012). Cancer therapy-induced cardiac toxicity in early breast cancer: addressing the unresolved issues. Circulation.

[11] Lang RM, Badano LP, Mor-Avi V (2015). Recommendations for cardiac chamber quantification by echocardiography in adults: an update from the American Society of Echocardiography and the European Association of Cardiovascular Imaging. J Am Soc Echocardiogr.

[12] van der Linde D, van Hagen I, Veen K, Zuetenhorst H, van Dalen B (2023). Global longitudinal strain: an early marker for cardiotoxicity in patients treated for breast cancer. Neth Heart J.

[13] Dobson R, Ghosh AK, Ky B (2021). BSE and BCOS guideline for transthoracic echocardiographic assessment of adult cancer patients receiving anthracyclines and/or trastuzumab. JACC CardioOncol.

[14] Keramida K, Farmakis D, Bingcang J (2019). Longitudinal changes of right ventricular deformation mechanics during trastuzumab therapy in breast cancer patients. Eur J Heart Fail.

[15] Tadic M, Cuspidi C, Hering D, Venneri L, Danylenko O (2017). The influence of chemotherapy on the right ventricle: did we forget something?. Clin Cardiol.

[16] Harrison A, Hatton N, Ryan JJ (2015). The right ventricle under pressure: evaluating the adaptive and maladaptive changes in the right ventricle in pulmonary arterial hypertension using echocardiography (2013 Grover Conference series). Pulm Circ.

[17] Haddad F, Hunt SA, Rosenthal DN, Murphy DJ (2008). Right ventricular function in cardiovascular disease, part I: Anatomy, physiology, aging, and functional assessment of the right ventricle. Circulation.

[18] Zhao R, Shu F, Zhang C (2020). Early detection and prediction of anthracycline-induced right ventricular cardiotoxicity by 3-dimensional echocardiography. JACC CardioOncol.

[19] Kjaer A, Hesse B (2001). Heart failure and neuroendocrine activation: diagnostic, prognostic and therapeutic perspectives. Clin Physiol.

[20] Weber M, Hamm C (2006). Role of B-type natriuretic peptide (BNP) and NT-proBNP in clinical routine. Heart.

[21] Lyon AR, López-Fernández T, Couch LS (2022). 2022 ESC Guidelines on cardio-oncology developed in collaboration with the European Hematology Association (EHA), the European Society for Therapeutic Radiology and Oncology (ESTRO) and the International Cardio-Oncology Society (IC-OS). Eur Heart J.

[22] Arciniegas Calle MC, Sandhu NP, Xia H (2018). Two-dimensional speckle tracking echocardiography predicts early subclinical cardiotoxicity associated with anthracycline-trastuzumab chemotherapy in patients with breast cancer. BMC Cancer.

[23] Boczar KE, Aseyev O, Sulpher J (2016). Right heart function deteriorates in breast cancer patients undergoing anthracycline-based chemotherapy. Echo Res Pract.

[24] Laufer-Perl M, Perelman-Gvili M, Sirota Dorfman S (2022). Prevalence of right ventricle strain changes following anthracycline therapy. Life (Basel).

[25] Grover S, Leong DP, Chakrabarty A (2013). Left and right ventricular effects of anthracycline and trastuzumab chemotherapy: a prospective study using novel cardiac imaging and biochemical markers. Int J Cardiol.

